# Molecular mechanism of the anti-inflammatory and skin protective effects of *Syzygium formosum* in human skin keratinocytes

**DOI:** 10.1007/s10068-023-01380-4

**Published:** 2023-09-19

**Authors:** Seung Hoon Lee, Nan-Young Lee, Seung-Hyeon Choi, Cheong-Hae Oh, Gun-Woo Won, Mahesh Prakash Bhatta, Ji Hyun Moon, Chang-gyu Lee, Jong Hun Kim, Jong-ll Park, Jong-Tae Park

**Affiliations:** 1 Department of Biochemistry, Research Institute for Medical Science, Chungnam National University School of Medicine, Daejeon, Korea; 2 CARBOEXPERT Inc., Daejeon, 34134 Korea; 3 Department of Food Science and Biotechnology, Sungshin Women’s University, Seoul, 01133 Republic of Korea; 4 Department of Food Science and Technology, Chungnam National University, Daejeon, 34134 Korea

**Keywords:** Human skin keratinocytes, MAPK, *Syzygium formosum*, NF-κB, Irradiation damage

## Abstract

Irradiation injury, especially caused by UVB, of the skin is one of the critical reasons for skin inflammation and damage. The present study aimed to explore the protective effect of *Syzygium formosum* leafy extract (SFLE) and its mechanism of action against UVB-induced damages of human keratinocytes. In this study, SFLE was prepared from 100 kg dried leaves using industrial-scale processes. We found that SFLE markedly reduced markers of the skin inflammation in UVB-induced pro-inflammatory cytokines. Only 2 μg/mL of SFLE exhibited significantly stronger anti-inflammatory effects than the fivefold concentration of positive control. Intriguingly, an anti-inflammatory enzyme, heme oxygenase-1 expression was significantly induced by SFLE treatment. MMP-3 and -9 were, but not MMP-1, significantly reduced. SFLE inhibited the expression of the MAPK pathway, resulting in a decrease on UVB-induced reactive oxygen species. In conclusion, SFLE can potentially be used to treat skin inflammatory diseases.

**Supplementary Information:**

The online version contains supplementary material available at 10.1007/s10068-023-01380-4.

## Introduction

Skin aging is a result of different factors, such as exposure to ultraviolet (UV) radiation, as well as physical and chemical agents (Parrado et al., 2019; Park et al., 2021). UVB radiation is known to cause various adverse effects on the skin, including but not limited to edema, erythema, hyperplasia, immunosuppression, inflammation, sunburn, skin photoaging, and photocarcinogenesis (Aquino et al., 2002). Among them, photoaging accounts for about 80% of skin aging (Kammeyer and Luiten, 2015).

The UV spectrum is divided into three distinct wavelengths, namely UVA (320–400 nm), UVB (290–320 nm) and UVC (200–290 nm). While UVC is absorbed into the ozone layer, UVA and UVB can reach the Earth’s surface (Cela et al, 2015). UVB irradiation is known to induce the production of inflammatory cytokines (Bashir et al., 2009; Ishida and Sakaguchi, 2007). As the results of UVB irradiation, erythema, edema and epidermal hyperplasia are often found on the skin (De Fabo and Noonan, 1983; El Ghiss et al., 2009). The role of keratinocytes in various responses to UVB-induced photo-damage is well established. (Bashir et al., 2009; Ishida and Sakaguchi, 2007). Therefore, treatment modalities that reduce or block the generation of inflammatory cytokines are important to prevent inflammatory diseases of the skin. When the skin is exposed to UVB irradiation, the inflammatory response to the degenerative process is mediated primarily by the overproduction of active oxygen species (ROS) in keratinocytes. ROS induces inflammation responses and serious DNA damages (Hanson and Clegg, 2002).

In particular, the activation of mitogen-activated protein kinases (MAPK) is critical for the generation of inflammatory cytokines by UVB irradiation ( Davis, 1993; Fisher et al., 1998; Karin and Hunter, 1995). The MAPK has three family members that include extracellular-signal-related protein (ERK), c-JUN-N-terminal kinases (JNK) and p38 MAPK (Kallunki et al., 1994). The MAPK signaling pathway participates in numerous cellular processes such as cell proliferation, development, cell cycle, morphogenesis, stress resistance, and intercellular signaling (Juretic et al., 2001; Yosimichi et al., 2001). This signaling pathway in turn activates a variety of extracellular matrix proteins including metrix metalloproteninases (MMP) -1, -3 and -9, which are known to be associated with skin senescence, breakdown of collagen and are thus related to aggravation of photoaging (Lee, 2021).

*Syzygium formosum* is an evergreen tree in the family Myrtaceae, extensively distributed in Southeast Asia, Brazil, Taiwan and India. *S. formosum* leaf has been traditionally used as food or tea to treat many inflammatory and immune disorder diseases. Its leafy extract (SFLE) has pharmacological activity such as anti-allergic, anti-bacterial activity, anti-inflammatory and antioxidant activity (Minh, 1968; Nong, 2009; Nguyen et al., 2018; Lee et al., 2006; Chang et al., 1999). Previous studies have reported that SFLE contained greater amount of biologically active phytochemicals than the well-known medicinal plant, *Centella aciatica*. Triterpenic acids were found as major bio-functional chemicals in SFLE (Park et al., 2021). Despite of the medicinal applications of the plant, only few studies on the anti-inflammation effects of SFLE have been reported. Furthermore, the mechanism of its anti-inflammatory actions has not yet been fully investigated.

In this study, we investigated the possible anti-inflammatory properties of SFLE on inflammatory cytokines in human skin keratinocytes that were induced by UVB radiation. In addition, to explore the potential mechanisms underlying the pharmacological effects of SFLE, we assessed the response of the MAPK signaling pathway and inflammatory regulators. The aim of this study was to explore how SFLE functions by assessing the levels of inflammatory cytokines, ROS, MAPK, HO-1 and MMPs in human skin keratinocytes.

## Materials and methods

### Preparation of SFLE and *C. asiatica* extract

A significant amount of *S. formosum* leaves (more than 100 kg of dry leaves) were collected from the suburbs of Hanoi, Vietnam. Additionally, 1 kg of dried *C. asiatica* was purchased from Jung Woo-dang (Seoul, Korea). Extraction of both plant materials was carried out using 70% ethanol at a ratio of 12-fold (v/w). The extraction process was performed under specific conditions as previously described [24]. The resulting extracts were analyzed for major triterpenic acids using liquid chromatography-mass spectrometry, following the protocol described by Park et al. (2021).

### Cell culture

The HaCaT cells, which are human skin keratinocytes were provided by the Korea Research Institute of Bioscience & Biotechnology (KRIBB, Daejeon, Republic of Korea). The cells were cultured in DMEM medium supplemented with 10% fetal bovine serum (FBS, WELGENE, Gyeongsan, Republic of Korea), streptomycin (100 μg/mL) and penicillin (100 units/mL) at 37 °C in a 5% CO_2_ environment.

### Cell viability assay

The HaCaT cells (1 × 10^5^ cells/mL) were seeded on 96-well plate and cultured overnight. SFLE was diluted with indicated concentration with medium and added in cell culture plate. The samples were incubated for 24 h, and then treated with an MTT solution according to standard procedures. The incubation continued for an additional hour before further analysis. After removing the supernatant, 0.1 mL of DMSO was added, and the optical density at 620 nm was measured using a microplate reader. (Epoch, Biotek, Winooski, VT, USA).

### Compound treatment and UVB irradiation

To perform the experiment, HaCaT cells were seeded at a concentration of 2 × 10^5^ cells/mL in 60 mm dish and incubated overnight. The cells were then treated with different concentrations of SFLE (2 and 5 μg/mL) and 10 μg/mL of *C. asiatica* extract as a positive control for 4 h. Subsequently, the cells were washed twice with 2 mL of Dulbecco’s Phosphate-Buffered Saline (DPBS, WELGENE, Gyeongbuk, Republic of Korea) and exposed to 10 mJ/cm2 of UVB. The cells were further incubated in serum-free medium (DMEM) containing the compound for 2 or 4 h.

### Reverse transcription polymerase chain reaction (RT-PCR)

The Trizol method was used to extract total RNA from HaCaT cells as per the manufacturer’s instructions (Invitrogen, Waltham, MA, USA). The extracted RNA was then subjected to reverse transcription using AccuPower® CycleScript™ RT PreMix (Bioneer, Daejeon, Republic of Korea) to produce cDNA. To assess the mRNA expression levels of the target genes, PCR analysis was performed. The results were normalized to the expression levels of the housekeeping gene, glyceraldehyde 3-phosphate dehydrogenase (GAPDH). The primer sequences utilized in this analysis can be found in Supplementary Table 2.

### Enzyme-linked immunosorbent assay (ELISA)

To determine the concentrations of inflammatory cytokines, the supernatants of HaCaT cells were collected and analyzed using an ELISA kit (BioLegend, San Diego, CA, USA) according to the manufacturer’s instructions. The cytokines measured included IL-6, IL-8 and TNF-α.

### Western blotting

Protein extraction from cultured HaCaT cells was performed using a protease inhibitor cocktail and cell lysis buffer (Cell Signaling Technology, MA, USA). The resulting homogenate was obtained after ultrasonic treatment and centrifugation at 10,000 rpm for 15 min at 4 °C. The sample was separated on a 12% SDS-polyacrylamide gel, and then transferred onto a PVDF membrane that had been blocked with 5% w/v fat-free dry milk in PBS containing 0.5% Tween-20. Primary antibodies, including actin (purchased from Santa Cruz Biotechnology, Dallas, USA), p-ERK (44-680G, ThermoFisher Scientific, MA, USA), ERK, p-JNK, JNK, p-p38, and p38 (all from ThermoFisher Scientific) were used to detect the target proteins.

### Measurement of ERK and JNK

To measure ROS levels in HaCaT cells, the cells were first homogenized in cell lysis buffer and then centrifuged at 10,000 rpm for 15 min at 4 °C. The resulting supernatant was used for the quantification of ROS levels using the DCF ROS/RNS Assay kit (Abcam, #238,535, Cambridge, UK), which employs the fluorogenic probe DCFHDiOxyQ that is specific for ROS/RNS. The assay was carried out according to the manufacturer’s instructions as described previously by Vandierendonck et al. (2021). In order to measure the levels of ROS/RNS in the sample, fluorescence intensity was detected at an excitation wavelength of 480 nm and an emission wavelength of 530 nm.

### Statistical analysis

The results are presented as mean ± standard error (SEM). Statistical analysis was performed using GraphPad Prism v.5.10 software (GraphPad Software Inc., San Diego, CA, USA), utilizing one-way analysis of variance (ANOVA) followed by post-hoc multiple comparison tests with Tukey’s HSD test. A p-value less than 0.05 was considered statistically significant, and all statistical comparisons between different treatments were evaluated using one-way ANOVA with the Tukey multiple comparison post-test.

## Results and discussion

### Effects of SFLE and UVB on the viability

**Fig. 1:**
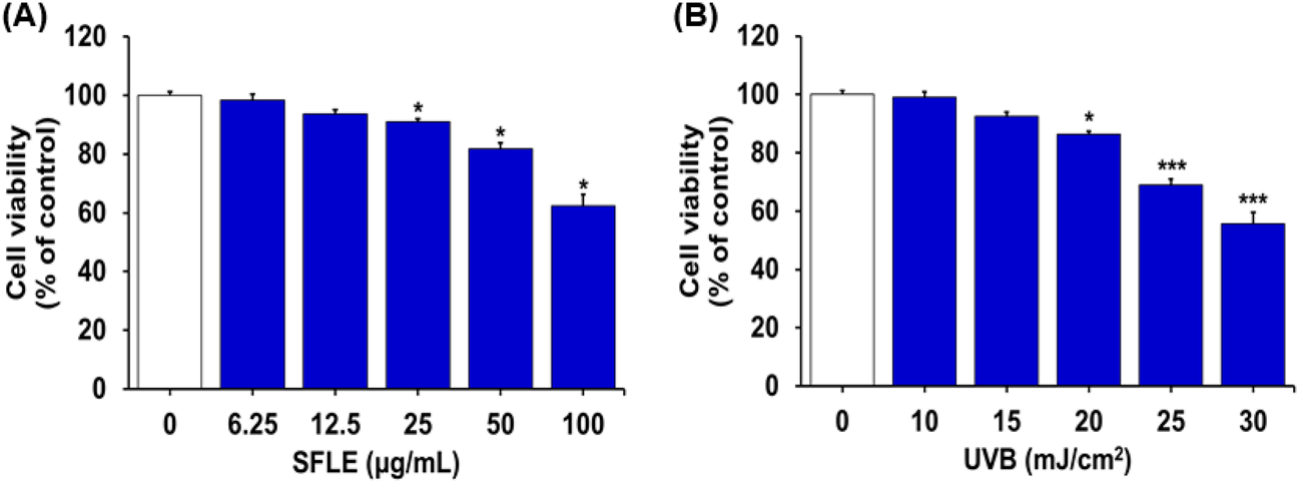
Effects of SFLE extract and UVB dose on the viability of HaCaT. (**A**) The cells were treated with indicated concentration (6.25–100 μg/mL) of SFLE for 24 h. (**B**) The cells were irradiated with various dose of UVB (10–30 mJ/cm^2^) then incubated in serum-free medium for 4 h. Cell viability was assessed by MTT assay. These results are expressed as the mean ± SEM of three independent experiment

Major triterpenes in the extracts are shown in Supplementary Table 1. Asiatic acid, corosolic acid and betulinic acids were major components in SFLE, but only asiatic acid and madecassic acid were detected in *C. asiatica* extract. Total triterpenic acids were tenfold higher in SFLE than *C. asiatica*. To identify the effect of SFLE on human skin keratinocytes cells, the cells were cultured with SFLE (0–100 μg/mL) and UVB (0–30 mJ/cm^2^) for 24 h, and cell viability was measured by MTT assay. SFLE and UVB were not cytotoxic to human skin keratinocyte cells at the concentrations used 2 and 5 μg/mL and 10 mJ/cm^2^, respectively (Fig. 1).

### Inhibitory effects of SFLE on UVB-induced inflammation

**Fig. 2:**
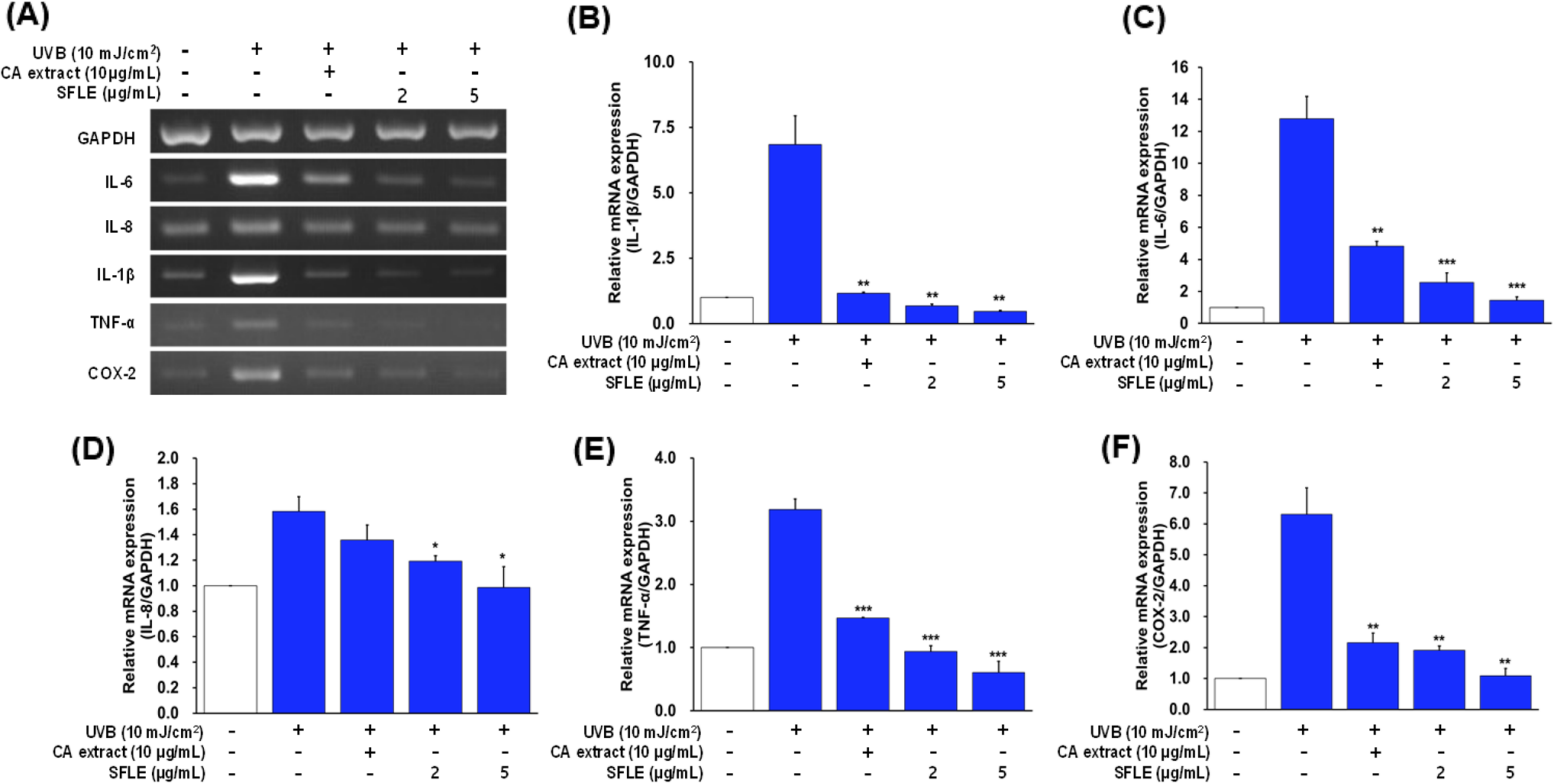
Inhibition of (**A**–**F**) inflammatory cytokines in UVB-induced HaCaT cells by treatment of SFLE. Cells were pretreated with SFLE for 4 h. *Centella asiatica* (CA) extract was used for comparison. Statistical significance: *, P < 0.05; **, P < 0.01; ***, P < 0.001 compared with the UVB irradiation group

To identify the inhibitory effects of SFLE on UVB-induced cytokines such as IL-1β, -6, -8, TNF-α and COX-2 in HaCaT keratinocytes, we measured pro-inflammatory cytokines levels using an ELSIA assay. Treatment with UVB significantly increased IL-1β, -6, -8, TNF-α and COX-2 levels compared with the control group, while treatment with 5 μg/mL SFLE decreased UVB-induced IL-1β, -6, -8, TNF-α and COX-2 (Fig. 2A, B). We next examined the effects in the critical inflammatory cytokine secretion such as IL-6, -8 and TNF-α on UVB treatment human skin keratinocytes. UVB-induced IL-6, -8 and TNF-α secretion were blocked by SFLE treatment in human skin keratinocytes (Fig. 2C). These results showed that SFLE treatment suppressed pro-inflammatory cytokines levels.

UVB radiation can have various effects on human health, with one of the most notable being the stimulation of vitamin D production in the skin. Vitamin D plays a crucial role in many physiological processes, including cell proliferation, bone growth, immune function and insulin secretion. A deficiency in vitamin D can lead to various health problems. Therefore, regular and minimum exposure to UVB is necessary to maintain optimal health (Holick, 2003). Despite its importance in stimulating vitamin D production, UVB radiation can also have numerous negative effects on the skin. Acute exposure to UVB can result in inflammation, sunburn, edema, erythema and immunosuppression. These biological effects of UVB on the skin can have significant impacts on human health (Afaq et al., 2005). Inflammatory infiltrating cells irradiated with UVB produce cytokines. In keratinocytes, function and regulation of cytokines have been extensively studied. The IL-6, IL-8 and TNF-α cytokines are known to be associated with the progression of UVB irradiation and are produced by keratinocytes. They have been linked to cellular damage and the development of inflammatory diseases. Studies suggest that SFLE may have anti-inflammatory properties by inhibiting these cytokines. Therefore, SFLE has the potential to be used as a treatment for skin inflammation (Ding et al., 2009; Kovaríková et al., 2004). These results suggest that SFLE has the ability to inhibit pro-inflammatory cytokines, which could make it a promising treatment option for skin inflammation. Overall, this study provides further evidence supporting the potential of SFLE as an anti-inflammatory agent for skin-related conditions.

### Inhibitory effects of SFLE on UVB-induced ROS

**Fig. 3:**
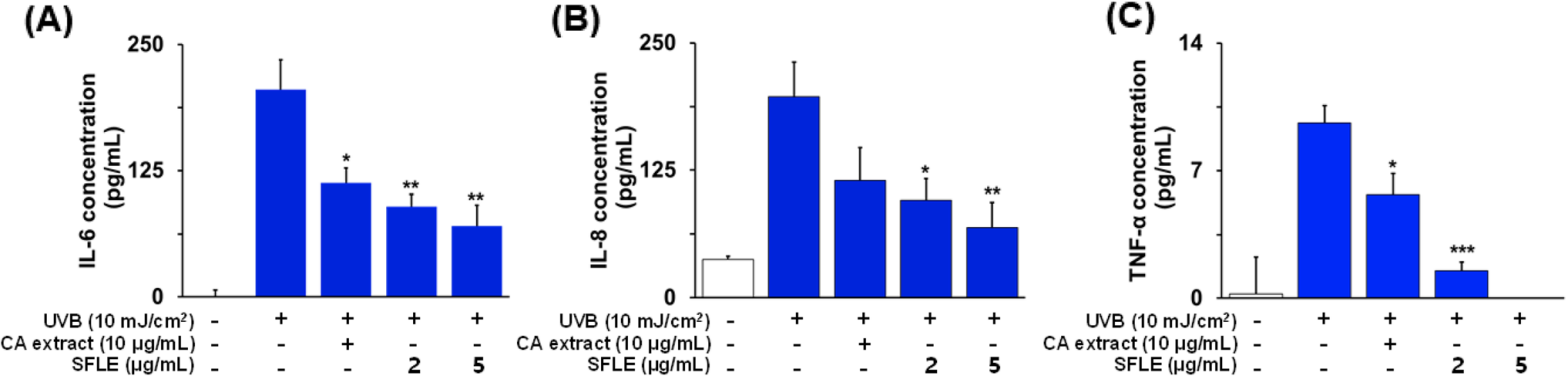
Repression of major inflammatory cytokines such as (**A**) IL-6, (**B**) IL-8 and (**C**) TNF-α in UVB-induced HaCaT cells by treating the plant extracts. SFLE were pretreated to the cells 4-h before UVB irradiation. CA indicates *Centella asiatica*. Statistical significance: *P < 0.05; **P < 0.01; ***P < 0.001 compared with the UVB irradiation group

To investigate the effects of SFLE on anti-oxidative enzymes in human skin keratinocytes, we measured the levels of heme oxygenase-1 (HO-1) in cells treated with SFLE and UVB. Our results showed that treatment with UVB significantly decreased HO-1 enzyme levels compared to control cells, but treatment with 5 μg/mL SFLE increased the levels of HO-1 that were reduced by UVB exposure (Fig. 3A, B). Additionally, we assessed the generation of reactive oxygen species using DCFH-DiOxyQ to determine whether SFLE treatment affected ROS levels. Our findings showed that treatment with UVB significantly increased ROS generation compared to control cells, while treatment with 5 μg/mL SFLE decreased UVB-induced ROS generation (Fig. 3c). These results suggest that SFLE treatment can suppress ROS generation in human skin keratinocytes.

### Inhibition effects of SFLE on UVB-induced matrix metalloproteinase

**Fig. 4:**
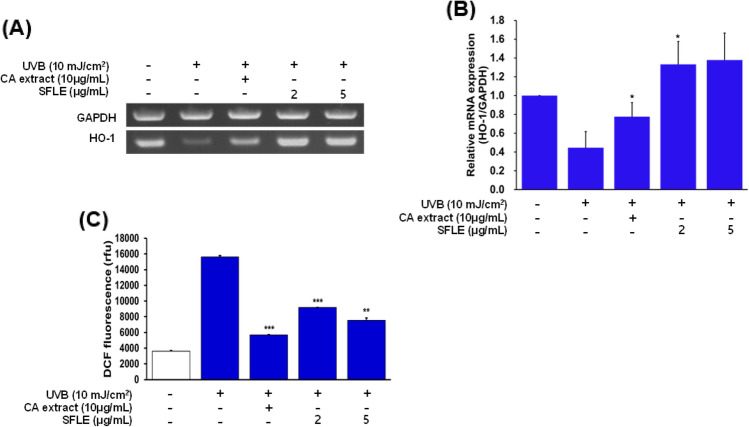
Anti-oxidative effect SFLE. Cells were pretreated with SFLE 4-h before UVB irradiation. Heme oxygenase-1 (HO-1) expression was measured (**A** and **B**) with the Quantification of ROS using DCFH-DiOxyQ (**C**). Statistical significance: *P < 0.05; **P < 0.01; ***P < 0.001 compared with the UVB irradiation group

In order to investigate the potential inhibitory effects of SFLE on MMPs in UVB-damaged human skin keratinocytes, we utilized RT-PCR to analyze the RNA levels of MMP-1, -3 and -9. SFLE decreased the MMP-3 and MMP-9 levels in human skin keratinocytes but did not decrease the MMP-1 levels (Fig. 4). These results showed that SFLE treatment decreased MMP-3 and -9 in human skin keratinocytes. One of the key characteristics of skin that has been affected by photo-aging is the disruption of the connective tissue and subsequent loss of structural integrity in the extracellular matrix. A crucial characteristic of photoaged skin is the impairment of the connective tissue and the loss of structural integrity in its extracellular matrix (ECM) (Yang et al., 2019). In order to maintain optimal hydration and elasticity in the skin, the ECM plays a crucial role in providing a network structure that contains essential structural proteins such as collagen and elastin (Birkedal-Hansen, 1987; Freedberg et al., 2001). MMP-1, a member of the MMP family, not only activates MMP-3 and degrades collagen type IV, but also degrades collagen fragments via MMP-9 (Kwon et al., 2019). In photoaging skin, changes to the composition of the ECM may occur due to high expression of MMPs (Anggakusuma and Hwang, 2010). Signaling molecules such as ERK, JNK and p38 MAPK play an essential role in transmitting extracellular signals into the nucleus to regulate gene expression (Yang et al., 2009). The family of MMPs, secreted by fibroblasts and keratinocytes, includes MMP-1, MMP-3 and MMP-9, and they play a critical role in the process of skin photoaging. Thus, high expression of MMP in photoaged skin may be a major cause of decreased skin elasticity and wrinkle formation. Our study found that treatment with SFLE significantly inhibited MMP-3 and MMP-9, suggesting that SFLE could potentially be used as a therapeutic option to improve the skin’s extracellular matrix.

### Inhibition effects of SFLE on UVB-induced MAPK pathway

**Fig. 5:**
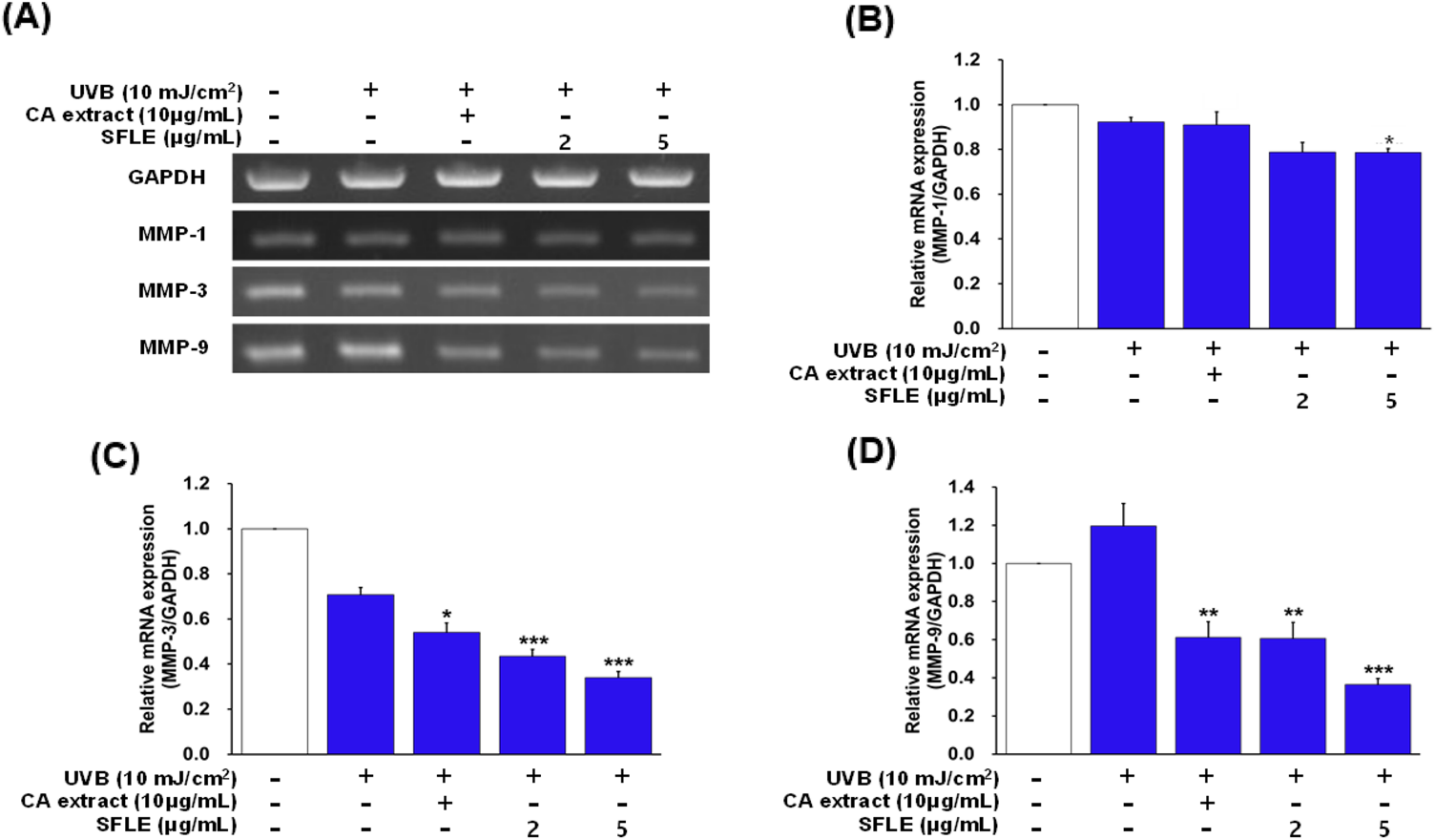
Repression of matrix-metalloproteinases (MMPs) by SFLE in HaCaT Cells. The cells were exposure with UVB 4-h after treatment of SFLE. Major MMPs such as (**A**–**D**) MMP-1, MMP-3 and MMP-9 were measured. Statistical significance: *P < 0.05; **P < 0.01; ***P < 0.001 compared with the UVB irradiation group

MAPK is a crucial signaling pathway involved in the regulation of transcriptional induction of inflammatory molecules. Therefore, the anti-inflammatory pathway suppressed against UVB irradiation by inhibiting MAPK activation. To evaluate the inhibitory effects of SFLE on UVB-induced MAPK signaling in HaCaT keratinocytes, we measured phosphorylation (p-) MAPK (ERK, JNK) using western blot and ELSIA analysis. Compared to control cells, exposure to UVB radiation resulted in a noteworthy elevation of phosphorylated ERK and JNK levels. However, treatment with 5 μg/mL SFLE was found to decrease the UVB-induced levels of phosphorylated MAPK ERK and JNK. This indicates the potential of SFLE to mitigate the effects of UVB-induced skin damage (Fig. 5). These results showed that SFLE treatment suppressed MAPK pathways.

### Skin protective mechanism of SFLE against UVB-induced damages

Our investigation represents the first attempt to elucidate the underlying inhibitory mechanism of SFLE on UVB-induced skin inflammation in human keratinocytes, offering novel insights into the potential therapeutic advantages of SFLE in the management of UVB-induced skin damage. We observed that SFLE effectively suppressed UVB-induced skin inflammation by downregulating the expression of pro-inflammatory cytokines (IL-1β, -6, -8 and TNF-α) via its modulation of MAPK signaling pathways. This provides important insights into the potential therapeutic applications of SFLE for treating UVB-induced skin damage.

**Fig. 6:**
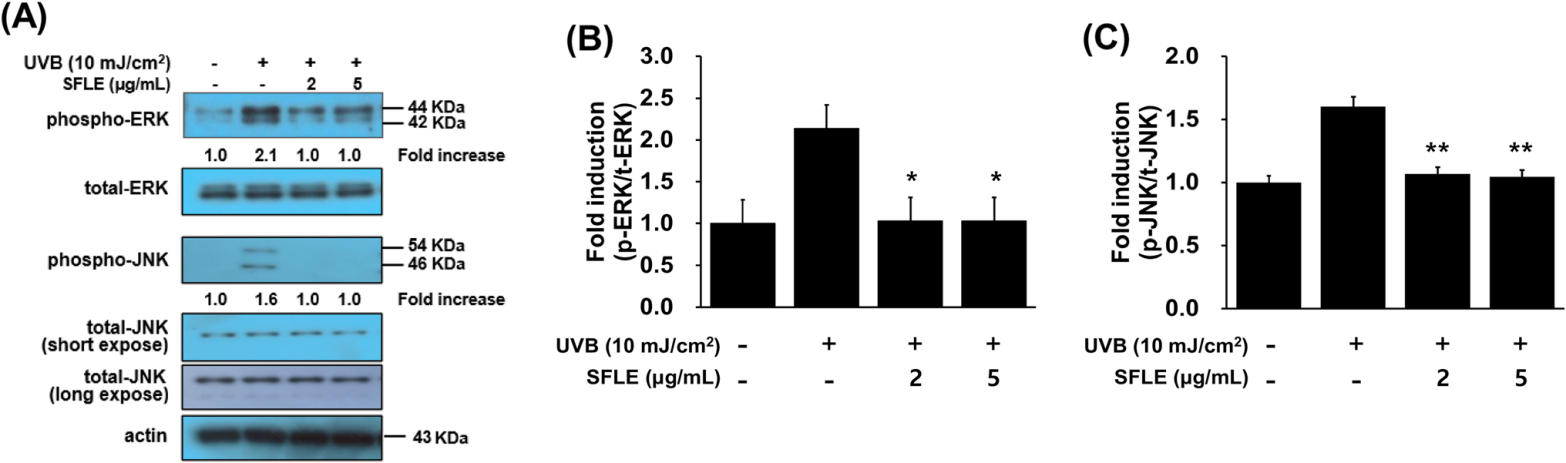
Inhibition of UVB-induced expression of (**A**–**C**) ERK and JNK by SFLE in HaCaT by pretreatment of SFLE. The cells were treated with SFLE before UVB irradiation. Statistical significance: *P < 0.05; **P < 0.01 compared with the UVB irradiation group

MAPK regulate important inflammation pathways following stimulation with UVB irradiation (Carlsen et al., 2004; Hong et al., 2009; Muthusamy and Piva, 2010). UVB radiation exposure can lead to the generation of ROS, which activate various cellular signaling pathways, including the MAPK signaling pathways. These pathways, including JNK, ERK and p38, play a critical role in the cellular response to UVB-induced ROS generation, which can mediate skin inflammatory responses and apoptosis (Leng et al., 2009; Neves et al., 2009). In epidermal keratinocytes, various extracellular stimuli, including UVB, can cause stress-related signal cascades, of which the most important is activation of MAPK. Given the ability of SFLE to scavenge ROS and inhibit inflammation, it is reasonable to speculate that many of the biological events triggered by UVB radiation may be mediated by SFLE. These findings highlight the potential of SFLE as a therapeutic agent for preventing or treating skin damage induced by UVB exposure. Among the signaling pathways stimulated by UVB, MAPKs is playing a key role by modulating UVB damaging effects. This study investigated the inhibitory effects of SFLE on UVB-induced MAPK activation, and confirmed its anti-inflammatory properties through its effects on MAPK signaling pathways. These findings shed light on the potential therapeutic applications of SFLE for preventing or treating UVB-induced skin damage. To investigate the potential impact of SFLE on the skin’s oxidative defense system, we assessed levels of the antioxidant enzyme HO-1. This enzyme is known to help repair skin damage by reducing the effects of free radicals, which are common contributors to photoaging. Additionally, we observed a significant decrease in the UVB-induced phosphorylation of ERK and JNK following treatment with SFLE. These findings suggest that SFLE may have potential as a therapeutic agent for preventing and treating skin damage caused by oxidative stress. HO-1 plays a critical role in the catabolism of heme, and its activation leads to the release of iron, biliverdin, and carbon monoxide. These findings provide valuable insights into the potential mechanisms by which SFLE may exert therapeutic effects against UVB-induced skin damage (Park et al., 2012). This study found that UVB exposure resulted in a significant decrease in HO-1 levels, while treatment with SFLE increased the levels of HO-1. Also, we investigated the impact of SFLE on UVB-induced ROS generation by measuring DCFH-DiOxyQ fluorescence in human skin keratinocytes in our study. Treatment with UVB significantly increased ROS generation compared with the control group, while treatment with 5 μg/mL SFLE decreased UVB-induced ROS generation. These findings suggest that SFLE suppresses the inflammation cytokines by inhibiting the MAPK signaling pathway, inhibit the ROS generation via oxidative defense protein. To our knowledge, this is the first study to examine the effects of SFLE on human skin keratinocytes, particularly regarding ROS production, anti-oxidant enzyme reduction, inflammatory cytokines, and matrix metalloproteinase-3 and -9, via the MAPK signaling pathways (Fig. 6). The SFLE has anti-inflammatory effect and be useful as therapeutic agent to relieve photoaging.

We found that SFLE treatment on UVB-irradiated human skin keratinocytes significantly attenuated the production of UVB-stimulated pro-inflammatory cytokines such as IL-1β, -6, -8, COX-2 and TNF-α. It is noteworthy that fivefold lower dose of SFLE (2 μg/mL) exhibited much stronger anti-inflammatory effects than *C. asiatica* extract. These inhibitory effects were exerted by the inhibition of inflammation-regulated ERK and JNK MAPK pathway signaling. We also found that SFLE inhibited the important matrix metalloproteinase in skin, MMP-3 and MMP-9. In addition, the inhibitory effects of SFLE on UVB-induced inflammation related molecules are associated with decrease of ROS and increase of HO-1. In conclusion, our results indicate that *S. formosum* leafy extract could be a feasible therapeutic ingredient to attenuate skin senescence via affecting inflammatory cytokines, ROS, MAPK, HO-1 and MMPs in human skin keratinocytes.

### Supplementary Information

Below is the link to the electronic supplementary material.

Supplementary file1 (DOCX 18 kb)
