# Supplementary material for: Molecular mechanism of the anti-inflammatory and skin protective effects of Syzygium formosum in human skin keratinocytes
Source: Food Sci Biotechnol. 2023 Sep 19;33(3):689–97. doi: 10.1007/s10068-023-01380-4 (PMC10805749; doi:10.1007/s10068-023-01380-4)
Supplement: Supplementary file 1 — Supplementary file1 (DOCX 18 kb) [file 10068_2023_1380_MOESM1_ESM.docx]

Supplementary Table 1 Major triterpenic acids compositions in *Syzygium formosum* leafy extract (SFLE) and *Centella asiatica* extract. SFLE was prepared in large scale extraction (>100 kg dried leaf).

| ***Samples*** | **Triterpenic acids^a^ (mg/g dry extract)** | | | | | | | **Total** |
| --- | --- | --- | --- | --- | --- | --- | --- | --- |
|  | **Madecassic**  **acid** | **Asiatic**  **acid** | **Maslinic**  **acid** | **Corosolic**  **acid** | **Betulinic**  **acid** | **Oleanolic**  **acid** | **Ursolic**  **acid** |  |
| **SFLE** | 2.17 ±  0.12 | 24.0 ± 0.67 | 5.20 ±  0.14 | 19.2 ±  0.50 | 18.6 ±  0.74 | 1.37 ±  0.05 | 2.86 ±  0.04 | 73.4 ±  1.5 |
| ***C. asiatica*** | 2.46 ±  0.1 | 4.23 ±  0.01 | ND^b^ | ND | ND | ND | ND | 6.70 ±  0.11 |

^a^ Analyses were done three times independently. Concentrations are expressed as mean ± standard deviation.

^b^ Not detected.

Supplementary Table 2 List of primers for PCR

| Gene | Forward | Reverse |
| --- | --- | --- |
| IL-6 | ATGAACTCCTTCTCCACAAGC | GTTTTCTGCCAGTGCCTCTTTG |
| IL-8 | TCTGTGTGAAGGTGCAGTT | AGCCCTCTTCAAAAACTTCT |
| IL-1β | AAACAGATGAAGTGCTCCTTCCAGG | TGGAGAACACCACTTGTTGCTCCA |
| TNF-α | GAGCTGAGAGATAACCAGCTGGTG | CAGATAGATGGGCTCATACCAGGG |
| COX-2 | CTTCTGCCTGACACCTTTC | TTCAAGGAGAATGGTGCTCC |
| MMP-1 | AGATTTGCCAAGAGCAGATG | AATTGTTGGTCCACCTTTCA |
| MMP-3 | TTCTCCAGGCTGTATGAAGG | TCAGTGTTGGCTGAGTGAAA |
| MMP-9 | CTTCCAAGGCCAATCCTACT | TCAAAGTTCGAGGTGGTAGC |
| HO-1 | AGTTGCTGTAGGGCTTTATG | CTCTGAAGTTTAGGCCATTG |
| GAPDH | AAGTGGATATTGTTGCCATC | ACTGTGGTCATGAGTCCTTC |
